# Entanglement Robustness via Spatial Deformation of Identical Particle Wave Functions

**DOI:** 10.3390/e23060708

**Published:** 2021-06-03

**Authors:** Matteo Piccolini, Farzam Nosrati, Giuseppe Compagno, Patrizia Livreri, Roberto Morandotti, Rosario Lo Franco

**Affiliations:** 1 Dipartimento di Ingegneria, Università di Palermo, Viale delle Scienze, 90128 Palermo, Italy; matteo.piccolini@unipa.it (M.P.); farzam.nosrati@unipa.it (F.N.); patrizia.livreri@unipa.it (P.L.); 2 INRS-EMT, 1650 Boulevard Lionel-Boulet, Varennes, QC J3X 1S2, Canada; roberto.morandotti@inrs.ca; 3 Dipartimento di Fisica e Chimica—Emilio Segrè, Università di Palermo, via Archirafi 36, 90123 Palermo, Italy; giuseppe.compagno@unipa.it

**Keywords:** entanglement protection, indistinguishable particles, open quantum systems

## Abstract

We address the problem of entanglement protection against surrounding noise by a procedure suitably exploiting spatial indistinguishability of identical subsystems. To this purpose, we take two initially separated and entangled identical qubits interacting with two independent noisy environments. Three typical models of environments are considered: amplitude damping channel, phase damping channel and depolarizing channel. After the interaction, we deform the wave functions of the two qubits to make them spatially overlap before performing spatially localized operations and classical communication (sLOCC) and eventually computing the entanglement of the resulting state. This way, we show that spatial indistinguishability of identical qubits can be utilized within the sLOCC operational framework to partially recover the quantum correlations spoiled by the environment. A general behavior emerges: the higher the spatial indistinguishability achieved via deformation, the larger the amount of recovered entanglement.

## 1. Introduction

It is well known that the environment of an open quantum system produces a detrimental noise that has to be dealt with during the implementation of many useful quantum information processing schemes [1,2]. One of the main goals in the development of fault-tolerant enhanced quantum technologies is to provide a strategy to protect the entanglement from such degradation. This challenge has been addressed, e.g., by the seminal works on quantum error corrections [3,4,5,6], structured environments with memory effects [7,8,9,10,11,12,13,14,15,16,17], distillation protocols [18,19,20], decoherence-free subspaces [21,22], dynamical decoupling and control techniques [23,24,25,26,27,28,29,30,31,32].

It is not unusual to find identical particles (i.e., subsystems such as photons, atoms, nuclei, electrons or any artificial qubits of the same species) as building blocks of quantum information processing devices and quantum technologies [33,34]. Nonetheless, the standard approach to identical particles based on unphysical labels is known to give rise to formal problems when trying to asses the correlations between constituents with (partially or completely) overlapping spatial wave functions [35,36]. For this reason, many alternative approaches have been developed to deal with the formal aspects of the entanglement of identical particles [36,37,38,39,40,41,42,43,44,45,46,47,48,49,50,51,52,53,54]. Among these, the *no-label* approach [51,52,53] provides many advantages: for example, it allows us to address the correlations between identical particles exploiting the same tools used for nonidentical ones (e.g., the von Neumann entropy of the reduced density matrix). Furthermore, it provides the known results for distinguishable particles in the limit of non-overlapping (spatially separated) wave functions. When treating the global multiparticle state as a whole indivisible object, in the no-label approach entanglement strictly depends on both the spatial overlap of the wave functions and on spatially localized measurements. An entropic measure has been recently introduced [55] to quantify the degree of indistinguishability of identical particles arising from their spatial overlap. Furthermore, an operational framework based on spatially localized operations and classical communication (sLOCC), where the no-label approach finds its natural application, has been firstly theorized [53] and later experimentally implemented [56,57] as a way of activating physical entanglement. Such framework has also been applied to fields such as the exploitation of the Hanbury Brown–Twiss effect with identical particles [58], quantum entanglement in one-dimensional systems of anyons [59], entanglement transfer in a quantum network [60] and quantum metrology [61,62]. Moreover, in a recent paper [55] it has been shown that spatial indistinguishability, even partial, can be exploited to recover the entanglement spoiled from the preparation noise of a depolarizing channel.

In this work, we aim to extend the results of Reference [55] to the wider scenario of different paradigmatic noise channels, namely amplitude damping, phase damping and depolarizing channels, under both Markovian and non-Markovian regimes. To do so, we introduce *spatial deformations*, i.e., transformations turning initially spatially separated (and thus distinguishable) particles into indistinguishable ones by making their wave functions spatially overlap. We then analyze the entanglement dynamics of two identical qubits interacting separately with their own environment, with the goal of showing that the application of the mentioned spatial deformation at a given time of the evolution, immediately followed by the sLOCC measurement, constitutes a procedure capable of recovering quantum correlations.

This paper is organized as follows: in Section 2 we introduce the general framework of the analyzed dynamics and the main tools used, namely the deformation operation and the sLOCC protocol. The main results follow in Section 3, where we describe the considered model and study the scenarios of an amplitude damping channel, a phase damping channel and a depolarizing channel. Finally, Section 4 summarizes and discusses the main results.

## 2. Materials and Methods

In this section we introduce the goal of this paper and the main tools used to achieve it.

**Figure 1:**
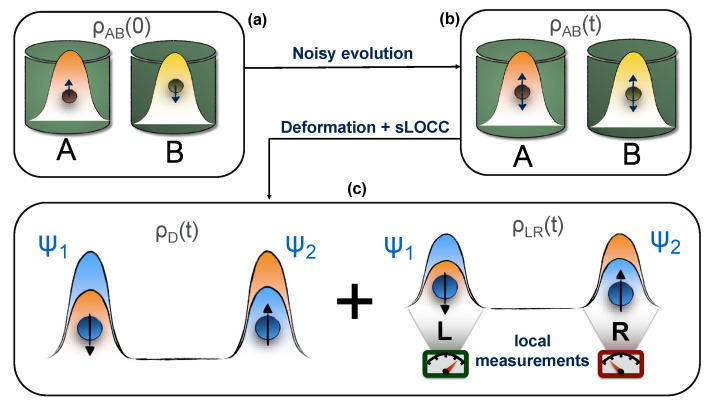
State evolution in the considered scenario. (**a**) The two qubits are initially prepared in the pure entangled state $\rho_{\mathrm{AB}}\left(0\right)$. (**b**) They are left to interact with a noisy environment, whose detrimental action produces the mixed state $\rho_{\mathrm{AB}}\left(t\right)$. (**c**) At time *t* a deformation of the two particles wave functions is performed, immediately followed by a sLOCC measurement.

Let us consider the following process, illustrated in Figure 1: at the beginning, two identical qubits in the entangled state $\rho_{\mathrm{AB}}\left(0\right)$ occupy two different regions of space *A* and *B*, thus being distinguishable and individually addressable. Here, they locally interact with two spatially separated and independent noisy environments that spoil the initial correlations. At time *t*, the two particles become decoupled from the environments and undergo a deformation, which makes their wave functions spatially overlap into the state $\rho_{D}\left(t\right)$. Immediately after that, a sLOCC measurement is performed to generate the entangled state $\rho_{\mathrm{LR}}\left(t\right)$. In this work, we show that this *deformation + sLOCC* procedure allows for the recovery of the entanglement spoiled by the previously introduced noise in an amount that depends on the degree of spatial indistinguishability achieved with the deformation. Three different models of environmental noise shall be considered: an amplitude damping channel, a phase damping channel and a depolarizing channel.

Notice that here the system–environment interaction occurs when the two particles are still distinguishable and no finite time interval separates the deformation from the immediately subsequent sLOCC operation. It will thus be interesting to compare the results of this work with those discussed in Reference [63], where the interaction with the noisy channels happens instead during a finite time interval between the deformation and the sLOCC operation, that is when the qubits are indistinguishable in the frame of the localized environments.

The deformation process bringing two particles to spatially overlap shall be now briefly introduced, followed by a recall of the sLOCC operational framework.

### 2.1. Deformations of Identical Particle States

Given a multipartite quantum system, a quantum transformation acting differently on each subpart changing the relations among them is called a *deformation*. In this section we focus on the specific set of continuous deformations that modify the single spatial wave functions of identical particles. In what follows, the no-label formalism [51] is used.

Let us take a non-entangled state of two identical particles $|\Phi \rangle =|\phi_{1},\phi_{2}\rangle$, where $\phi_{i}$ (i=1,2) is identified by the values of a complete set of commuting observables describing a spatial wave function $\psi_{i}$ and an internal degree of freedom $\tau_{i}$. We suppose that the two particles are initially spatially separated, e.g., localized in two distinct regions *A* and *B* such that $|\psi_{1}^{\left(0\right)}\rangle =|A\rangle$, $|\psi_{2}^{\left(0\right)}\rangle =|B\rangle$ and 〈A|B〉=0. We want to modify the spatial wave functions of the two particles in order to make them overlap. Thus, we introduce a deformation D such that

(1) $$|\phi_{1},\phi_{2}\rangle =|A\tau_{1}\rangle ⊗|B\tau_{2}\rangle \overset{D}{\to }|\psi_{1}\tau_{1},\psi_{2}\tau_{2}\rangle ,$$

where $\psi_{1}$ and $\psi_{2}$ are now at least partially overlapped. Since the two spatially overlapping particles are also identical, they are now *indistinguishable*: their final global state cannot be written as the tensor product of single particle states anymore and must be considered as a whole, i.e., $|\psi_{1}\tau_{1},\psi_{2}\tau_{2}\rangle \neq |\psi_{1}\tau_{1}\rangle ⊗|\psi_{2}\tau_{2}\rangle$ [51,52]. Notice that the expression above, applied to an elementary pure state of two identical particles, is valid for both bosons and fermions.

A deformation operator acting on identical particles is not, in general, unitary, and its normalized action on a state ρ written in terms of a convex set of density matrices $\left\{\rho_{i}\right\}$, $\rho =\sum_{i}p_{i}\;\rho_{i}$ with $p_{i}\in \left[0,1\right]$ and $\sum_{i}p_{i}=1$, is thus

(2) $$D\left[\rho \right]=\frac{D\rho D^{†}}{\mathrm{Tr}[DD^{†}\rho ]}=\sum_{i}{\bar{p}}_{i}D\left[\rho_{i}\right],$$

where

(3) $${\bar{p}}_{i}=\frac{\mathrm{Tr}[DD^{†}\rho_{i}]}{\mathrm{Tr}[DD^{†}\rho ]},\;\;\;D\left[\rho_{i}\right]=\frac{D\rho_{i}D^{†}}{\mathrm{Tr}[DD^{†}\rho_{i}]}.$$

Each two-particle state $\rho_{i}$ is, in general, a given combination of elementary basis states and the post-deformation state D[ρ] naturally encompasses the selection rules associated to the bosonic or fermionic statistics.

### 2.2. sLOCC, Spatial Indistinguishability, Concurrence and Fidelity

The natural extension of the standard local operation and classical communication framework (LOCC) for distinguishable particles to the scenario of indistinguishable (and thus individually unaddressable) particles is provided by the *spatially localized operations and classical communication* (sLOCC) environment [53]. Given a set of indistinguishable particles, sLOCC consist in a projective measurement of the global state over distinct spatially separated regions, followed by a post-selection of the outcomes where only one particle is found in each location. The result of this operation is an entangled state whose physical accessibility has been demonstrated in a quantum teleportation experiment [56].

Suppose we are given a state ρ of two identical and indistinguishable particles, e.g., obtained by the application (2) of the deformation (1), and assume they have pseudo-spin 1/2. The whole sLOCC operation (projection and post-selection) amounts to projecting the two qubits state on the subspace spanned by the basis

(4) $$B_{LR}=\left\{|L↑,R↑\rangle ,|L↑,R↓\rangle ,|L↓,R↑\rangle ,|L↓,R↓\rangle \right\},$$

via the projection operator

(5) $${\hat{\Pi }}_{LR}=\sum_{\sigma ,\tau =↑,↓}|L\sigma ,R\tau \rangle \langle L\sigma ,R\tau |.$$

Since the constituents are indistinguishable before the detection, it is impossible to know exactly which particle will be found in which region. The sLOCC operation generates the (normalized) two-particle entangled state

(6) $$\rho_{\mathrm{LR}}\left(t\right)=\frac{{\hat{\Pi }}_{LR}\;\rho \left(t\right)\;{\hat{\Pi }}_{LR}}{\mathrm{Tr}\left[{\hat{\Pi }}_{LR}\;\rho \right]},$$

with probability

(7) $$P_{\mathrm{LR}}=\mathrm{Tr}\left[{\hat{\Pi }}_{LR}\;\rho \right].$$

After the sLOCC measurement, the two qubits occupy two distinct regions of space and are thus now distinguishable and individually addressable. Furthermore, since in the no-label formalism the inner product between two-particle states is given by the rule [51]

(8) $$\langle \phi_{1}^{\prime },\phi_{2}^{\prime }|\phi_{1},\phi_{2}\rangle =\langle \phi_{1}^{\prime }|\phi_{1}\rangle \langle \phi_{2}^{\prime }|\phi_{2}\rangle +\eta \langle \phi_{1}^{\prime }|\phi_{2}\rangle \langle \phi_{2}^{\prime }|\phi_{1}\rangle ,$$

with η=1 for bosons and η=−1 for fermions, particle statistics naturally emerges within the sLOCC framework and is thus expected to play a role in the dynamics.

The sLOCC scenario also allows for the introduction of an entropic measure of the particles’ indistinguishability after the deformation (1), which depends on the achieved spatial distribution of their wave functions $\psi_{1},\;\psi_{2}$ over the two regions *L* and *R* where sLOCC measurement occurs. Given the probability $P_{X\psi_{i}}$ of finding the qubit having wave function $\psi_{i}$ (i=1,2) in the region *X* (X=L,R), the spatial indistinguishability measure is given by [55]

(9) $$I=-\frac{P_{L\psi_{1}}P_{R\psi_{2}}}{Z}\log_{2}\frac{P_{L\psi_{1}}P_{R\psi_{2}}}{Z}-\frac{P_{L\psi_{2}}P_{R\psi_{1}}}{Z}\log_{2}\frac{P_{L\psi_{2}}P_{R\psi_{1}}}{Z},$$

where $Z=P_{L\psi_{1}}P_{R\psi_{2}}+P_{L\psi_{2}}P_{R\psi_{1}}$. Notice that (9) ranges from 0 for spatially separated (thus distinguishable) particles (e.g., when $P_{L\psi_{1}}=P_{R\psi_{2}}=1$) to 1 for maximally indistinguishable particles ($P_{L\psi_{1}}=P_{L\psi_{2}},\;P_{R\psi_{1}}=P_{R\psi_{2}}$). Hereafter, we assume for convenience that the spatial wave functions of the single indistinguishable particles after the deformation have the form

(10) $$|\psi_{1}\rangle =l|L\rangle +r|R\rangle ,\;|\psi_{2}\rangle =l^{\prime }|L\rangle +r^{\prime }|R\rangle ,$$

where

(11) $$l=\langle L|\psi_{1}\rangle ,\;r=\langle R|\psi_{1}\rangle ,\;l^{\prime }=\langle L|\psi_{2}\rangle ,\;r^{\prime }=\langle R|\psi_{2}\rangle$$

are complex coefficients such that ${\left|l\right|}^{2}+{\left|r\right|}^{2}=|l^{\prime }{|}^{2}+{\left|r^{\prime }\right|}^{2}=1$. In the following analysis, we shall conveniently set $l=r^{\prime }$ to assure that the sLOCC probability $P_{\mathrm{LR}}$ is different from zero.

As previously stated, the state $\rho_{\mathrm{LR}}$ obtained by the sLOCC measurement is entangled. Among the existing entanglement quantifiers [35,64,65,66], we address the quantification of the quantum correlations characterizing the bipartite quantum state $\rho_{\mathrm{LR}}$ of two distinguishable qubits via the Wootters concurrence for convenience, namely [55,67]

(12) $$C\left(\rho_{\mathrm{LR}}\right)=\max\left\{0,\sqrt{\lambda_{4}}-\sqrt{\lambda_{3}}-\sqrt{\lambda_{2}}-\sqrt{\lambda_{1}}\right\},$$

where $\lambda_{i}$ are the eigenvalues in decreasing order of the matrix $\xi =\rho_{\mathrm{LR}}\;{\tilde{\rho }}_{\mathrm{LR}}$, with ${\tilde{\rho }}_{\mathrm{LR}}=\left(\sigma_{y}^{L}⊗\sigma_{y}^{R}\right)\;\rho_{\mathrm{AB}}^{*}\;\left(\sigma_{y}^{L}⊗\sigma_{y}^{R}\right)$ and $\sigma_{y}^{L}$, $\sigma_{y}^{R}$ being the usual Pauli matrix $\sigma_{y}$ localized, respectively, on the particle in L and in R.

Albeit our main interest relies in the amount of exploitable entanglement recovered after sLOCC, we also consider the fidelity [68] $F\left(\rho_{0},\rho_{\mathrm{LR}}\right)={\left(\mathrm{Tr}\;\sqrt{\sqrt{\rho_{0}}\;\rho_{\mathrm{LR}}\;\sqrt{\rho_{0}}}\right)}^{2}$ as a figure of merit to quantify the closeness between the post-processing state $\rho_{\mathrm{LR}}$ and the initial state $\rho_{0}$ of the system. Notice that if the initial state is pure, i.e., $\rho_{0}=|\psi_{0}\rangle \langle \psi_{0}|$, then the fidelity takes the simple form

(13) $$F(\rho_{0},\rho_{\mathrm{LR}})=\langle \psi_{0}\left|\rho_{\mathrm{LR}}\right|\psi_{0}\rangle .$$

## 3. Indistinguishability as a Feature for Recovering Entanglement

In this section we report our main results. Each of the two independent environments is modeled as a bath of harmonic oscillators in the vacuum state except for one mode, which is coupled to the qubit interacting with it. Considering a qubit-cavity model with just one excitation overall allows us to treat the reservoir as characterized by a Lorentzian spectral density [69,70]

(14) $$J\left(\omega \right)=\frac{\gamma }{2\pi }\frac{\lambda^{2}}{{\left(\omega -\omega_{0}\right)}^{2}+\lambda^{2}},$$

where $\omega_{0}$ is the qubit transition frequency, γ is the microscopic system–environment coupling constant related to the decay of the excited state of the qubit in the Markovian limit of a flat spectrum, and λ is the spectral width of the coupling quantifying the leakage of photons through the cavity walls. The relaxation time $\tau_{R}$ on which the state of the system changes is related to the coupling constant by the relation $\tau_{R}\approx \gamma^{-1}$, while the reservoir correlation time $\tau_{B}$ is connected to the spectral width of the coupling by $\tau_{B}\approx \lambda^{-1}$. These coefficients regulate the behavior of the system: when $\gamma <\lambda /2\;(\tau_{R}>2\tau_{B})$ the system is weakly coupled to the environment, the reservoir correlation time is shorter than the relaxation time and we are in a Markovian regime; when $\gamma >\lambda /2\;(\tau_{R}<2\tau_{B})$ instead, we are in the strong coupling scenario, where the relaxation time is shorter than the bath correlation time and the regime is non-Markovian. The way each qubit interacts with its own reservoir depends on the type of noise channel taken into account.

The action of the three noisy channels considered in this paper shall be computed within the usual Kraus operators formalism, or operator-sum representation [71]. The general expression of the single-qubit evolved density matrix is then given by $\rho \left(t\right)=\sum_{i}E_{i}\rho \left(0\right)E_{i}^{†}$, where the $E_{i}$’s are the time-dependent Kraus operators corresponding to the specific channel and depend on the disturbance probability (decoherence function) p(t). Each channel in fact introduces a time-dependent disturbance on the system with a probability p(t)=1−q(t) obtained by solving the differential equation [69,72]

(15) $$\dot{q}\left(t\right)=-\int_{0}^{t}dt_{1}\;f\left(t-t_{1}\right)\;q\left(t_{1}\right),$$

where the correlation function $f(t-t_{1})$ is given by the Fourier transform of the spectral density J(ω) of the reservoir, namely

(16) $$f\left(t-t_{1}\right)=\int d\omega \;J\left(\omega \right)\;e^{-i\left(\omega -\omega_{0}\right)\left(t-t_{1}\right)}.$$

Solving Equation (15) for the spectral density (14), one obtains the disturbance (or error) probability [69]

(17) $$p\left(t\right)=1-e^{-\lambda t}{\left[\cos\left(\frac{d\;t}{2}\right)+\frac{\lambda }{d}\sin\left(\frac{d\;t}{2}\right)\right]}^{2},$$

with $d=\sqrt{2\gamma \lambda -\lambda^{2}}$. Notice that this solution takes into account both Markovian and non-Markovian regimes, depending on the ratio λ/γ. In particular, in the Markovian limit of flat spectrum, which occurs for γ/λ≪1, it is straightforward to see that $p\left(t\right)=1-e^{-\gamma t/2}$, as expected [71]. In general, the error probability (17) is such that p(0)=0 and $\lim_{t\to \infty }\;p\left(t\right)=1$.

### 3.1. Amplitude Damping Channel

The amplitude damping channel is one of the most used models describing energy dissipation in quantum systems. This is mainly due to the wide range of physical phenomena that it encompasses, from the spontaneous emission of a photon by an atom [73,74,75] to processes involving spin chains [76], the scattering of a photon in cavity QED [71], superconducting qubits in circuit QED [77,78] and high temperature spin systems relaxing to the equilibrium state with their environment [71]. Furthermore, it can be easily simulated using linear-optics devices [79], thus making it of experimental interest also in the context of quantum photonics.

The action of the amplitude damping channel on a single qubit in the operator-sum representation is given by the Kraus operators [71]

(18) $$\begin{array}{c} E_{0}=|↑\rangle \langle ↑|+\sqrt{1-p(t)}\;|↓\rangle \langle ↓|=E_{0}^{†},\\ E_{1}=\sqrt{p(t)}\;|↑\rangle \langle ↓|,\;E_{1}^{†}=\sqrt{p(t)}\;|↓\rangle \langle ↑|. \end{array}$$

Consider two identical qubits initially prepared in the Bell singlet state

(19) $${|1_{-}\rangle }_{\mathrm{AB}}=\frac{1}{\sqrt{2}}\left(|A↑,B↓\rangle -|A↓,B↑\rangle \right),$$

with *A* and *B* being two distinct spatial regions (〈A|B〉=0). Thanks to the fact that the the two environmental interactions are independent, the state after the noisy interaction is given by

(20) $$\begin{array}{cc} \rho_{\mathrm{AB}}\left(t\right)= & \left(E_{0}^{A}⊗E_{0}^{B}\right)\rho_{\mathrm{AB}}\left(0\right)\left(E_{0}^{A}⊗E_{0}^{B}\right)\\ + & \left(E_{1}^{A}⊗E_{1}^{B}\right)\rho_{\mathrm{AB}}\left(0\right)\left(E_{1}^{A\;†}⊗E_{1}^{B\;†}\right)\\ + & \left(E_{0}^{A}⊗E_{1}^{B}\right)\rho_{\mathrm{AB}}\left(0\right)\left(E_{0}^{A}⊗E_{1}^{B\;†}\right)\\ + & \left(E_{1}^{A}⊗E_{0}^{B}\right)\rho_{\mathrm{AB}}\left(0\right)\left(E_{1}^{A\;†}⊗E_{0}^{B}\right), \end{array}$$

where $E_{i}^{X}$ (i=1,2,X=A,B) denotes the *i*-th single particle Kraus operator of Equation (18) acting on the qubit localized in region *X*, while $\rho_{\mathrm{AB}}\left(0\right)={|1_{-}\rangle }_{\mathrm{AB}}{\langle 1_{-}|}_{\mathrm{AB}}$ is the initial density matrix. Using Equation (18) in the above equation, one then finds

(21) $$\rho_{\mathrm{AB}}\left(t\right)=\left(1-p(t)\right){|1_{-}\rangle }_{\mathrm{AB}}{\langle 1_{-}|}_{\mathrm{AB}}+p\left(t\right)|A↑,B↑\rangle \langle A↑,B↑|.$$

We now want to apply the deformation defined in Equation (1) to the state (21) at time *t*. State ${|1_{-}\rangle }_{\mathrm{AB}}$ gets mapped to

(22) $${|{\bar{1}}_{-}\rangle }_{D}=\frac{1}{\sqrt{2}}\left(|\psi_{1}↑,\psi_{2}↓\rangle -|\psi_{1}↓,\psi_{2}↑\rangle \right),$$

which is not a normalized state since $\langle \psi_{1}|\psi_{2}\rangle \neq 0$. In order to write it in terms of a normalized state ${|{\bar{1}}_{-}\rangle }_{N}$, we compute

(23) $${\langle {\bar{1}}_{-}|{\bar{1}}_{-}\rangle }_{D}=C_{1}^{2},\;C_{1}:=\sqrt{1-\eta {\left|\langle \psi_{1}|\psi_{2}\rangle \right|}^{2}},$$

and write it as

(24) $${|{\bar{1}}_{-}\rangle }_{D}=C_{1}{|{\bar{1}}_{-}\rangle }_{N}.$$

The same is done for the deformation of |A↑,B↑〉, which gets mapped to

(25) $${|\psi_{1}↑,\psi_{2}↑\rangle }_{D}=C_{2}{|\psi_{1}↑,\psi_{2}↑\rangle }_{N},\;C_{2}:=\sqrt{1+\eta {\left|\langle \psi_{1}|\psi_{2}\rangle \right|}^{2}},$$

where

(26) $${\langle \psi_{1}↑,\psi_{2}↑|\psi_{1}↑,\psi_{2}↑\rangle }_{N}=1.$$

The normalized state resulting from the spatial deformation (2) of the state (21) is thus

(27) $$\rho_{D}\left(t\right)=\frac{\left(1-p(t)\right)C_{1}^{2}{|{\bar{1}}_{-}\rangle }_{N}{\langle {\bar{1}}_{-}|}_{N}+p\left(t\right)\;C_{2}^{2}{|\psi_{1}↑,\psi_{2}↑\rangle }_{N}{\langle \psi_{1}↑,\psi_{2}↑|}_{N}}{\left(1-p(t)\right)C_{1}^{2}+p\left(t\right)\;C_{2}^{2}}.$$

Following the scheme shown in Figure 1, we perform the sLOCC measurement immediately after the deformation, applying the projection operator (5) onto the state (27), which finally gives

(28) $$\rho_{\mathrm{LR}}\left(t\right)=\frac{\left(1-p(t)\right){\left|lr^{\prime }-\eta \;l^{\prime }r\right|}^{2}{|1_{-}\rangle }_{\mathrm{LR}}{\langle 1_{-}|}_{\mathrm{LR}}+p\left(t\right)\;{\left|lr^{\prime }+\eta \;l^{\prime }r\right|}^{2}|L↑,R↑\rangle \langle L↑,R↑|}{\left(1-p(t)\right){\left|lr^{\prime }-\eta \;l^{\prime }r\right|}^{2}+p\left(t\right)\;{\left|lr^{\prime }+\eta \;l^{\prime }r\right|}^{2}}$$

where $l,r,l^{\prime },r^{\prime }$ are the wave function coefficients defined in (11).

In order to study the entanglement evolution of the state $\rho_{\mathrm{LR}}\left(t\right)$ of Equation (28), we calculate the concurrence defined in Equation (12), which is

(29) $$C\left(\rho_{\mathrm{LR}}\left(t\right)\right)=\frac{{\left|lr^{\prime }-\eta \;l^{\prime }r\right|}^{2}\left(1-p(t)\right)}{{\left|lr^{\prime }-\eta \;l^{\prime }r\right|}^{2}\left(1-p(t)\right)+{\left|lr^{\prime }+\eta \;l^{\prime }r\right|}^{2}\;p\left(t\right)},$$

where the statistics parameter η explicitly appears, as expected. As a first consideration, we notice that the results about entanglement dynamics for bosons can be obtained from the ones for fermions (and vice versa) by simply changing sign to one of the coefficients $l,\;r,\;l^{\prime },\;r^{\prime }$ (that is, by shifting the phase of one of them by π). Therefore, in order to fix a framework to analyze the concurrence, we assume we are dealing with fermions whose spatial wave functions are distributed over the regions *L* and *R* with positive real coefficients. This reasoning shall hold for the other noisy channels, so that the presented results are also valid for bosons. With this assumption, we obtain the concurrence as

(30) $$C\left(\rho_{\mathrm{LR}}\left(t\right)\right)=\frac{\left[{\left(lr^{\prime }\right)}^{2}+{\left(l^{\prime }r\right)}^{2}+2\;ll^{\prime }rr^{\prime }\right]\left(1-p(t)\right)}{{\left(lr^{\prime }\right)}^{2}+{\left(l^{\prime }r\right)}^{2}+2\;ll^{\prime }rr^{\prime }\left(1-2p(t)\right)}.$$

**Figure 2:**
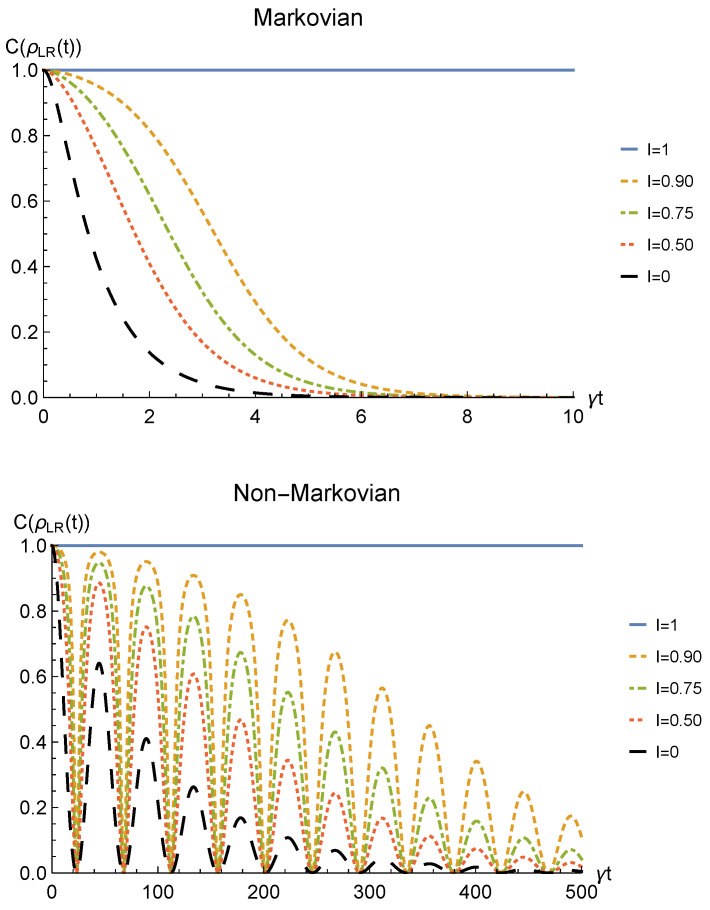
Concurrence of two identical qubits (fermions with $l,l^{\prime },r,r^{\prime }>0$, bosons with one of these four coefficients negative) in the initial state ${|1_{-}\rangle }_{\mathrm{AB}}$ subjected to localized amplitude damping channels, undergoing an instantaneous deformation+sLOCC operation at time *t* for different degrees of spatial indistinguishability I (with $\left|l|=\right|r^{\prime }|$). Both the Markovian (λ=5γ) (upper panel) and non-Markovian (λ=0.01γ) (lower panel) regimes are reported.

**Figure 3:**
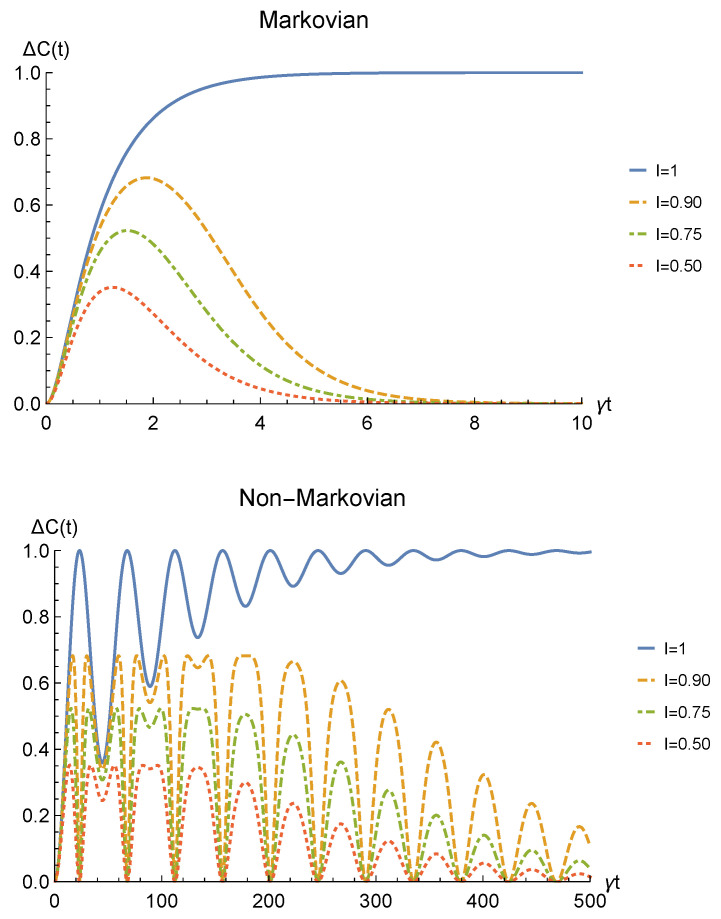
Net gain in the entanglement recovery of two identical qubits (fermions with $l,l^{\prime },r,r^{\prime }>0$, bosons with one of these four coefficients negative) in the initial state ${|1_{-}\rangle }_{\mathrm{AB}}$ under localized amplitude damping channels, thanks to the deformation+sLOCC operation performed at time *t*. Results are reported for different degrees of spatial indistinguishability I (with $\left|l|=\right|r^{\prime }|$). Both the Markovian (λ=5γ) (upper panel) and non-Markovian (λ=0.01γ) (lower panel) regimes are shown.

We point out that when no deformation is performed and the particles remain distinguishable in two distinct regions (I=0), the sLOCC projector (5) is equivalent to the identity operator. This implies that, when the particles are not brought to spatially overlap, our procedure gives the same entanglement we would have without performing the sLOCC operation. For this reason, we take the results for I=0 (black dashed lines in the following figures) as the term of comparison to quantify the entanglement gained due to the *deformation + sLOCC procedure*, i.e., $\Delta C\left(t\right):=C\left(\rho_{\mathrm{LR}}\left(t\right)\right)-C\left(\rho_{\mathrm{AB}}\left(t\right)\right)$. Figure 2 shows the concurrence (30) for both the Markovian and the non-Markovian regimes, while Figure 3 displays ΔC(t).

As can be seen in Figure 2, spatial indistinguishability (9) has a direct influence on the general behavior: when the particles are not perfectly indistinguishable (I≠1), the entanglement vanishes with a monotonic decay in the Markovian regime and with a periodic one in the non-Markovian regime. From Figure 3, we can see that when I≠1 the deformation and sLOCC procedure becomes inefficient in recovering the correlations as time grows. Nonetheless, it is interesting to notice that it provides an initial effective advantage as a consequence of the fact that the decay rate shown in Figure 2 lowers as the indistinguishability increases. However, when the particles wave functions maximally overlap (I=1, blue solid line), the entanglement remains stable at its initial maximum value, thus becoming unaffected by the noise. These results show that, in the scenario of the amplitude damping channel, we have provided an operational framework where spatial indistinguishability, even imperfect, of two identical qubits can be exploited as a scheme to recover quantum correlations spoiled by a short-time interaction with the noisy environment.

Finally, to check whether such procedure would be of any practical interest we have to analyze its theoretical probability of success. This strictly depends on the probability for the sLOCC projection (6) to produce a non-null result, physically representing a state that is not discarded during the postselection. Such probability is defined in Equation (7) and, for identical qubits undergoing a local interaction with an amplitude damping channel, it is equal to

(31) $$P_{\mathrm{LR}}\left(t\right)=\frac{{\left(lr^{\prime }\right)}^{2}+{\left(l^{\prime }r\right)}^{2}-2\eta \;ll^{\prime }rr^{\prime }\left(1-2\;p(t)\right)}{C_{1}^{2}\left(1-p(t)\right)+C_{2}^{2}\;p\left(t\right)}.$$

**Figure 4:**
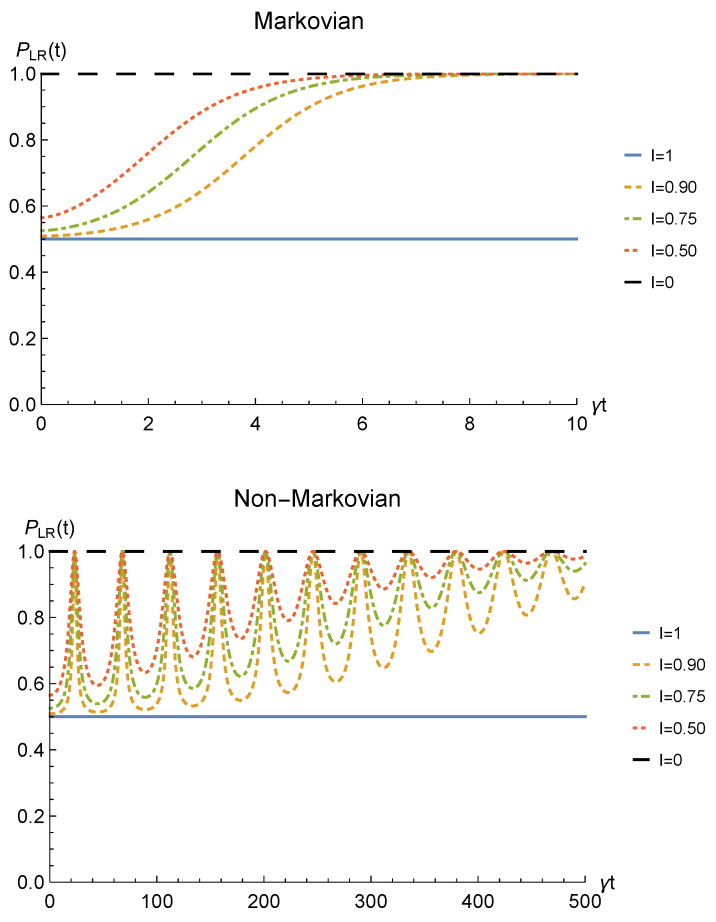
Success probability of obtaining a nonzero outcome from the sLOCC projection for fermions ($l,l^{\prime },r,r^{\prime }>0$ and $l=r^{\prime }$) interacting with localized amplitude damping channels. Different degrees of spatial indistinguishability are reported in both the Markovian (λ=5γ) (upper panel) and non-Markovian (λ=0.01γ) (lower panel) regimes.

Figure 4 shows the success probability (31) for different degrees of spatial indistinguishability in both the Markovian and non-Markovian regime in the case of fermions. As can be seen, when the indistinguishability is not maximum, the probability of success tends to 1 as time passes in both regimes, thus giving rise to a trade-off with the concurrence. The trade-off is confirmed by the probability being constant and equal to 1/2 when the concurrence is maximum, i.e., for I=1 (blue solid line). For bosons, the time-dependent success probability corresponding to I=1 (with the constraint $l=r^{\prime }=l^{\prime }=-r$) and to the concurrence plotted in Figure 2 is $P_{\mathrm{LR}}\left(t\right)=1-p\left(t\right)$ (notice, however, that this success probability can be improved by differently setting the coefficients of the spatial wave functions).

**Figure 5:**
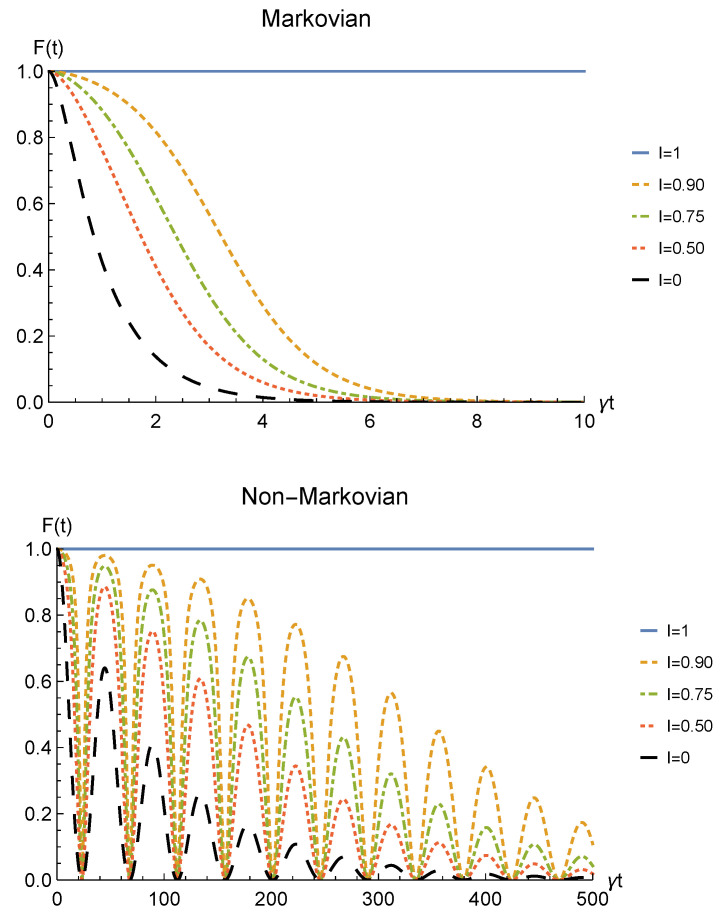
Fidelity of two identical qubits (fermions with $l,l^{\prime },r,r^{\prime }>0$) subjected to localized amplitude damping channels, computed between the initial state ${|1_{-}\rangle }_{\mathrm{LR}}{\langle 1_{-}|}_{\mathrm{LR}}$ and the state produced by an instantaneous deformation+sLOCC operation at time *t* for different degrees of spatial indistinguishability I (with $\left|l|=\right|r^{\prime }|$). Both the Markovian (λ=5γ) (upper panel) and non-Markovian (λ=0.01γ) (lower panel) regimes are reported.

We conclude the analysis of the amplitude damping channel by showing the fidelity (13) between the state of Equation (28) resulting from the sLOCC measurement and the initial state of Equation (19) where locations *A* and *B* are assumed to coincide with *L* and *R*, namely $\rho_{0}={|1_{-}\rangle }_{\mathrm{LR}}{\langle 1_{-}|}_{\mathrm{LR}}$. This is reported for fermions with real and positive coefficients in Figure 5 as a function of time and for different values of indistinguishability. Similarly to the concurrence, the fidelity decays to zero with time for I≠1, with a decay rate which diminishes with the spatial indistinguishability. When the maximal spatial indistinguishability is achieved, instead, the fidelity maintain its maximum value F=1 (I=1, solid blue line). This behavior holds in both the Markovian and non-Markovian regimes. These plots thus evidence how this procedure also enables state robustness with respect to the initially prepared state of the system.

### 3.2. Phase Damping Channel

The phase damping channel is used to model the inherently quantum non-dissipative physical situation where a system undergoes a loss of coherence without losing energy. In this scenario, the energy eigenstates of the system are not changed by the dynamics, but they accumulate a phase that is responsible for the gradual degradation of the interference terms. Physical systems undergoing this phenomena are, e.g., random telegraph noise and phase noisy lasers [80,81,82,83,84,85], photons randomly scattering through waveguides [86] and superconducting qubits under low-frequency noise [87].

A phase damping channel acting on a single qubit is described by the Kraus operators

(32) $$\begin{array}{c} E_{0}=|↑\rangle \langle ↑\rangle +\sqrt{1-p(t)}|↓\rangle \langle ↓|=E_{0}^{†},\\ E_{1}=\sqrt{p(t)}|↓\rangle \langle ↓|=E_{1}^{†}. \end{array}$$

Once again, we consider the Bell state ${|1_{-}\rangle }_{\mathrm{AB}}$ of two identical qubits defined in (19) as our initial state. The evolved state $\rho_{\mathrm{AB}}\left(t\right)$ after the interaction with the two independent environments is computed as in Equation (20), which for the phase damping channel described by the above Kraus operators gives

(33) $$\rho_{\mathrm{AB}}\left(t\right)=\left(1-\frac{p(t)}{2}\right){|1_{-}\rangle }_{\mathrm{AB}}{\langle 1_{-}|}_{\mathrm{AB}}+\frac{p(t)}{2}{|1_{+}\rangle }_{\mathrm{AB}}{\langle 1_{+}|}_{\mathrm{AB}},$$

where ${|1_{+}\rangle }_{\mathrm{AB}}$ is the Bell state defined as

(34) $${|1_{+}\rangle }_{\mathrm{AB}}=\frac{1}{\sqrt{2}}\left(|A↑,B↓\rangle +|A↓,B↑\rangle \right).$$

At time *t*, deformation (1) is applied to the state (33) to make the two particles spatially overlap. Deformation of ${|1_{-}\rangle }_{\mathrm{AB}}$ gives the state (22), while ${|1_{+}\rangle }_{\mathrm{AB}}$ gets mapped to

(35) $${|{\bar{1}}_{+}\rangle }_{D}=\frac{1}{\sqrt{2}}\left(|\psi_{1}↑,\psi_{2}↓\rangle +|\psi_{1}↓,\psi_{2}↑\rangle \right).$$

Once again, state ${|{\bar{1}}_{+}\rangle }_{D}$ is not normalized: it is indeed easy to show that

(36) $${|{\bar{1}}_{+}\rangle }_{D}=C_{2}{|{\bar{1}}_{+}\rangle }_{N},$$

where ${\langle {\bar{1}}_{+}|{\bar{1}}_{+}\rangle }_{N}=1$ and $C_{2}$ is defined in (25). Thus, the global normalized state after the deformation is

(37) $$\rho_{D}\left(t\right)=\frac{\left(1-\frac{1}{2}\;p\left(t\right)\right)C_{1}^{2}\;{|{\bar{1}}_{-}\rangle }_{N}{\langle {\bar{1}}_{-}|}_{N}+\frac{1}{2}\;p\left(t\right)\;C_{2}^{2}\;{|{\bar{1}}_{+}\rangle }_{N}{\langle {\bar{1}}_{+}|}_{N}}{\left(1-\frac{1}{2}\;p\left(t\right)\right)C_{1}^{2}+\frac{1}{2}\;p\left(t\right)\;C_{2}^{2}}.$$

Finally, the sLOCC operation is performed: the action of the projection operator (5) on the state (37), as defined in Equation (6), gives

(38) $$\begin{array}{cc} \rho_{\mathrm{LR}}\left(t\right) & =\frac{\left(1-\frac{1}{2}\;p\left(t\right)\right){\left|lr^{\prime }-\eta \;l^{\prime }r\right|}^{2}{|1_{-}\rangle }_{\mathrm{LR}}{\langle 1_{-}|}_{\mathrm{LR}}+\frac{1}{2}\;p\left(t\right){\left|lr^{\prime }+\eta \;l^{\prime }r\right|}^{2}{|1_{+}\rangle }_{\mathrm{LR}}{\langle 1_{+}|}_{\mathrm{LR}}}{\left(1-\frac{1}{2}\;p\left(t\right)\right){\left|lr^{\prime }-\eta \;l^{\prime }r\right|}^{2}+\frac{1}{2}\;p\left(t\right){\left|lr^{\prime }+\eta \;l^{\prime }r\right|}^{2}}. \end{array}$$

We now study the entanglement evolution of such a state by the concurrence $C(\rho_{\mathrm{LR}}\left(t\right))$, which is readily found to be

(39) $$\begin{array}{c} C\left(\rho_{\mathrm{LR}}\left(t\right)\right)=\max\left\{0,\;\lambda_{1}\left(t\right)-\lambda_{2}\left(t\right)\right\},\\ \lambda_{1}\left(t\right):=\max\left\{\lambda_{A}\left(t\right),\;\lambda_{B}\left(t\right)\right\},\lambda_{2}\left(t\right):=\min\left\{\lambda_{A}\left(t\right),\;\lambda_{B}\left(t\right)\right\}, \end{array}$$

with

$$\lambda_{A}\left(t\right):=\frac{\left(1-\frac{1}{2}\;p\left(t\right)\right){\left|lr^{\prime }-\eta \;l^{\prime }r\right|}^{2}}{\left(1-\frac{1}{2}\;p\left(t\right)\right){\left|lr^{\prime }-\eta \;l^{\prime }r\right|}^{2}+\frac{1}{2}\;p\left(t\right){\left|lr^{\prime }+\eta \;l^{\prime }r\right|}^{2}},$$

$$\lambda_{B}\left(t\right):=\frac{\frac{1}{2}\;p\left(t\right)\;{\left|lr^{\prime }+\eta \;l^{\prime }r\right|}^{2}}{\left(1-\frac{1}{2}\;p\left(t\right)\right){\left|lr^{\prime }-\eta \;l^{\prime }r\right|}^{2}+\frac{1}{2}\;p\left(t\right){\left|lr^{\prime }+\eta \;l^{\prime }r\right|}^{2}}.$$

Focusing the analysis once again on fermions with real and positive coefficients $l,r,l^{\prime },r^{\prime }$ to fix a framework, concurrence (39) is then equal to

(40) $$C\left(\rho_{\mathrm{LR}}\left(t\right)\right)=\frac{\left(1-p(t)\right)\left[{\left(lr^{\prime }\right)}^{2}+{\left(l^{\prime }r\right)}^{2}\right]+2ll^{\prime }rr^{\prime }}{{\left(lr^{\prime }\right)}^{2}+{\left(l^{\prime }r\right)}^{2}+\left(1-p(t)\right)2ll^{\prime }rr^{\prime }}.$$

**Figure 6:**
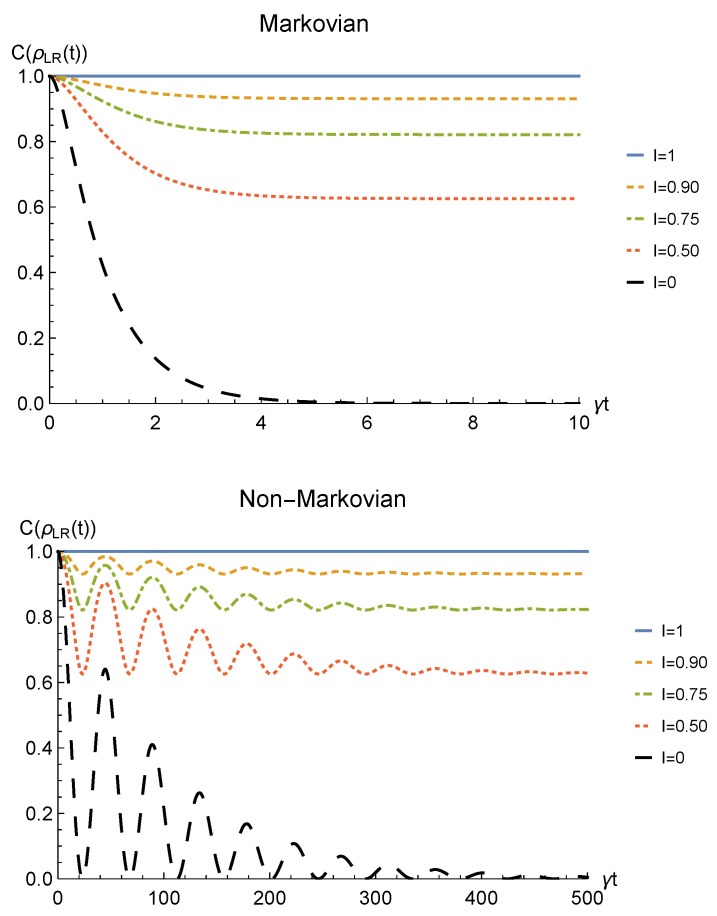
Concurrence of two identical qubits (fermions with $l,l^{\prime },r,r^{\prime }>0$, bosons with one of these four coefficients negative) in the initial state ${|1_{-}\rangle }_{\mathrm{AB}}$ interacting with localized phase damping channels, undergoing an instantaneous deformation+sLOCC operation at time *t* for different degrees of spatial indistinguishability I (with $\left|l|=\right|r^{\prime }|$). Both the Markovian (λ=5γ) (upper panel) and non-Markovian (λ=0.01γ) (lower panel) regimes are reported.

**Figure 7:**
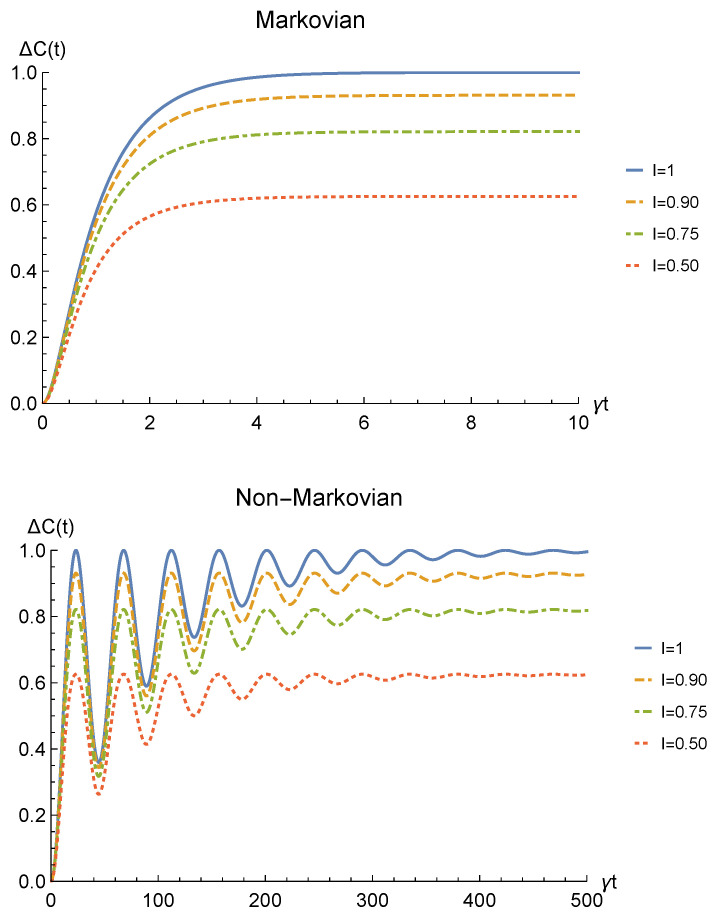
Net gain in the entanglement recovery of two identical qubits (fermions with $l,l^{\prime },r,r^{\prime }>0$, bosons with one of these four coefficients negative) in the initial state ${|1_{-}\rangle }_{\mathrm{AB}}$ under localized phase damping channels, thanks to the deformation+sLOCC operation performed at time *t*. Results are reported for different degrees of spatial indistinguishability I (with $\left|l|=\right|r^{\prime }|$). Both the Markovian (λ=5γ) (upper panel) and non-Markovian (λ=0.01γ) (lower panel) regimes are shown.

The time behavior of the concurrence of Equation (40) is plotted in Figure 6 for both the Markovian and the non-Markovian regime, while the net gain due to the deformation and sLOCC operation is depicted in Figure 7. Once again, the entanglement recovered is found to decrease as the interaction time increases where the generated spatial indistinguishability is not maximum. As in the amplitude damping scenario, such dephasing is monotonic in the Markovian regime and periodic in the non-Markovian one, with a decay rate that decreases as particle indistinguishability increases. Nonetheless, differently from that case, the entanglement now does not vanish. Indeed, for t→∞ it reaches a constant value which, under the above assumptions, is given by

(41) $$C_{\infty }=\frac{2ll^{\prime }rr^{\prime }}{{\left(lr^{\prime }\right)}^{2}+{\left(l^{\prime }r\right)}^{2}}.$$

Furthermore, when the indistinguishability is maximum (I=1, blue solid line) quantum correlations after the sLOCC measurement are completely immune to the action of the noisy environment and maintain their initial value. Is is important to highlight that the existence of such a steady value for the entanglement of identical particles is only due to the spatial indistinguishability of the qubits and to the procedure used to produce the entangled state, i.e., the sLOCC operation. This result clearly shows that spatial indistinguishability of identical qubits can be exploited to recover quantum correlations spoiled by the detrimental noise of a phase damping-like environment interacting independently with the constituents, as shown in Figure 7.

Finally, the success (sLOCC) probability of obtaining the outcome $\rho_{\mathrm{LR}}\left(t\right)$ for two identical qubits undergoing local phase damping channels is

(42) $$P_{\mathrm{LR}}\left(t\right)=\frac{{\left(lr^{\prime }\right)}^{2}+{\left(l^{\prime }r\right)}^{2}-2\eta \;ll^{\prime }rr^{\prime }\left(1-p(t)\right)}{\left(1-\frac{1}{2}p\left(t\right)\right)C_{1}^{2}+\frac{1}{2}p\left(t\right)C_{2}^{2}}.$$

**Figure 8:**
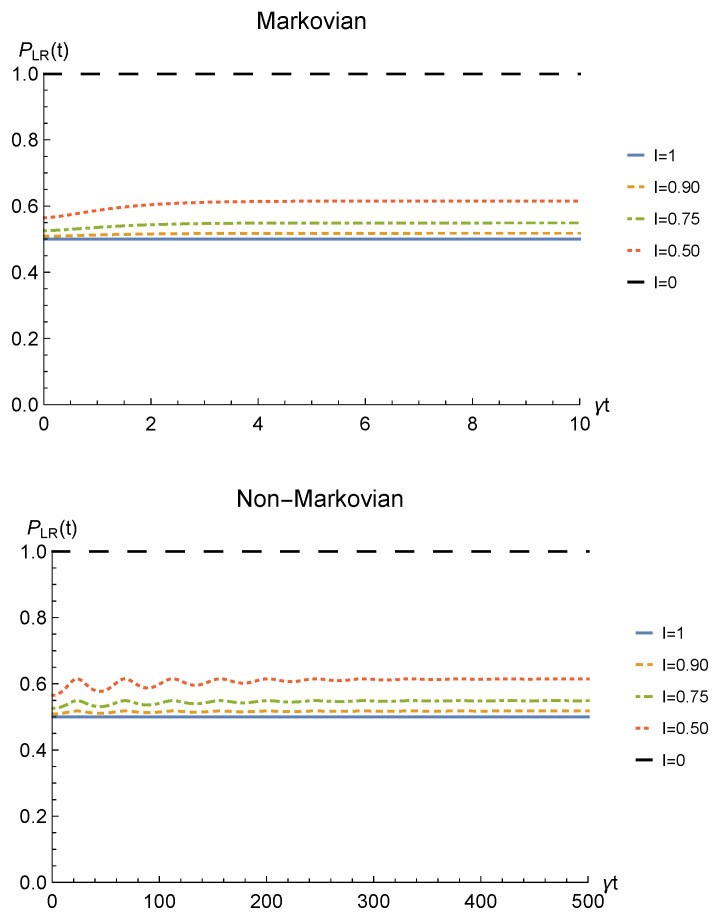
Probability of obtaining a non-zero outcome from the sLOCC projection for fermions (with $l,l^{\prime },r,r^{\prime }>0$ and $l=r^{\prime }$) interacting with localized phase damping channels. Different degrees of spatial indistinguishability are reported in both the Markovian (λ=5γ) (upper panel) and non-Markovian (λ=0.01γ) (lower panel) regimes.

Figure 8 depicts the behavior of the sLOCC probability of success (7) for fermions (with real and positive coefficients of the spatial wave functions) for different values of I. Once again, there is a trade-off between the probability of success and the concurrence, with $P_{\mathrm{LR}}\left(t\right)=1$ when the particles are distinguishable (black dashed line) and $P_{\mathrm{LR}}=1/2$ for perfectly indistinguishable qubits (blue solid line). A similar general behavior is found for bosons (with the constraint $l=r^{\prime }=l^{\prime }=-r$), having $P_{\mathrm{LR}}\left(t\right)=1-p\left(t\right)/2$ in the case of maximal indistinguishability I=1.

**Figure 9:**
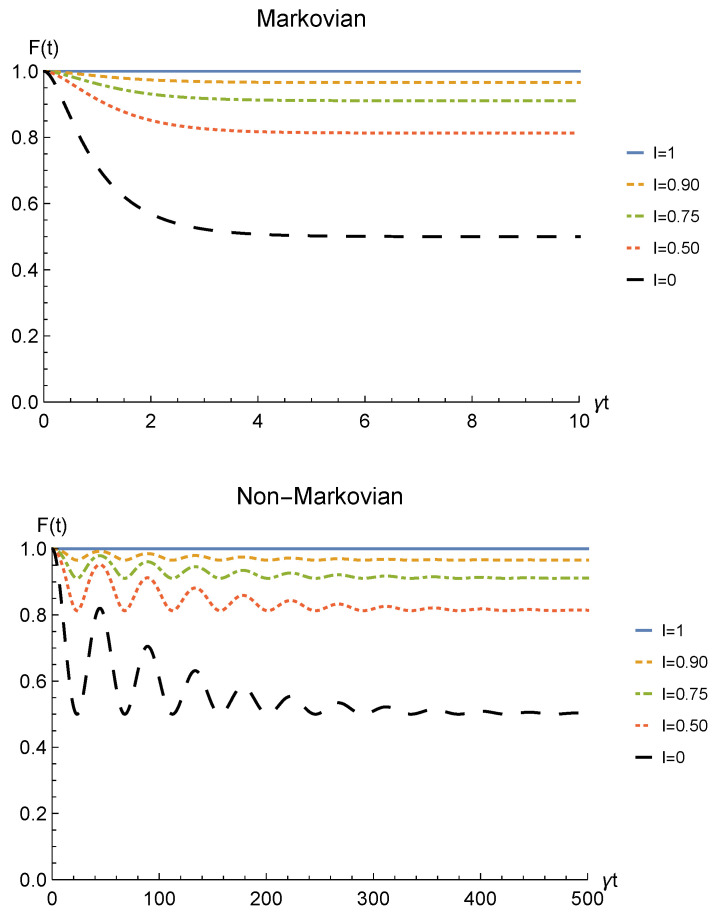
Fidelity of two identical qubits (fermions with $l,l^{\prime },r,r^{\prime }>0$) interacting with localized phase damping channels, computed between the initial state ${|1_{-}\rangle }_{\mathrm{LR}}{\langle 1_{-}|}_{\mathrm{LR}}$ and the state produced by an instantaneous deformation+sLOCC operation at time *t* for different degrees of spatial indistinguishability I (with $\left|l|=\right|r^{\prime }|$). Both the Markovian (λ=5γ) (upper panel) and non-Markovian (λ=0.01γ) (lower panel) regimes are reported.

The same general relation between concurrence and spatial indistinguishability is found also for the fidelity between the initial state $\rho_{0}={|1_{-}\rangle }_{\mathrm{LR}}{\langle 1_{-}|}_{\mathrm{LR}}$ and the final one (38), displayed in Figure 9 for both the Markovian and the non-Markovian regime.

Notice that, differently from the amplitude damping scenario, this time the fidelity does not vanish but it reaches an asymptotic value that increases with the indistinguishability, starting from F=1 for distinguishable particles (I=0, black dashed line) and reaching the maximum value F=1 when I=1 (solid blue line). An efficient state robustness is then activated by the proposed procedure for pure dephasing noise.

### 3.3. Depolarizing Channel

In this section we reconsider and expand the results on entanglement protection at the preparation stage presented in Reference [55].

The depolarizing channel describes the process where a system undergoes a symmetric decoherence [71]. This type of noise can occur, for instance, during an isotropic interaction of a spin-1/2-like particle (qubit) with a bosonic or spin-like environment [88,89,90,91]. The depolarizing process can be encountered in nuclear magnetic resonance setups [92,93] and Bose–Einstein condensates [94,95], where the decoherence process is typically caused by a residual fluctuating magnetic field. Traveling photons can also undergo a depolarizing noise, due to optical scattering when photons become randomly polarized [96,97,98].

A depolarizing channel acting on a system of two qubits has the effect of leaving it untouched with probability 1−p(t) and of introducing a white noise that drives it into the maximally mixed state with probability p(t). This is, for instance, a typical noise occurring when quantum states are initialized. Supposing once again that our system of two identical particles is initially in the Bell state ${|1_{-}\rangle }_{\mathrm{AB}}$, it is well known that this kind of noisy interaction produces the Werner state [71]

(43) $$\rho_{\mathrm{AB}}\left(t\right)=W_{\mathrm{AB}}^{-}\left(t\right):=\left(1-p(t)\right){|1_{-}\rangle }_{\mathrm{LR}}{\langle 1_{-}|}_{\mathrm{LR}}+\frac{1}{4}\;p\left(t\right)\;1\;\;1,$$

where 11 is the 4×4 identity operator. Hereafter, we work for convenience on the Bell states basis

$$B_{B}=\left\{{|1_{+}\rangle }_{\mathrm{AB}},{|1_{-}\rangle }_{\mathrm{AB}},{|2_{+}\rangle }_{\mathrm{AB}},{|2_{-}\rangle }_{\mathrm{AB}}\right\},$$

where ${|1_{+}\rangle }_{\mathrm{AB}},\;{|1_{-}\rangle }_{\mathrm{AB}}$ have been previously defined respectively in (19) and (34), while ${|2_{+}\rangle }_{\mathrm{AB}}$ and ${|2_{-}\rangle }_{\mathrm{AB}}$ are given by

(44) $$\begin{array}{c} {|2_{+}\rangle }_{\mathrm{AB}}=\frac{1}{\sqrt{2}}\left(|A↑,B↑\rangle +|A↓,B↓\rangle \right),\\ {|2_{-}\rangle }_{\mathrm{AB}}=\frac{1}{\sqrt{2}}\left(|A↑,B↑\rangle -|A↓,B↓\rangle \right). \end{array}$$

We recall that since such basis is orthonormal, the identity operator can be written as

$$1\;\;1=\sum_{\begin{array}{c} j=1,2\\ s=↑,↓ \end{array}}{|j_{s}\rangle }_{\mathrm{AB}}{\langle j_{s}|}_{\mathrm{AB}}.$$

At time *t* we deform the two qubits wave functions. The deformation of states ${|1_{+}\rangle }_{\mathrm{AB}}$ and ${|1_{-}\rangle }_{\mathrm{AB}}$ has already been discussed in (35) and (22), while states ${|2_{+}\rangle }_{\mathrm{AB}}$ and ${|2_{-}\rangle }_{\mathrm{AB}}$ are mapped respectively to

(45) $${|{\bar{2}}_{+}\rangle }_{D}=C_{2}{|{\bar{2}}_{+}\rangle }_{N},\;{|{\bar{2}}_{-}\rangle }_{D}=C_{2}{|{\bar{2}}_{-}\rangle }_{N},$$

where ${\langle {\bar{2}}_{+}|{\bar{2}}_{+}\rangle }_{N}={\langle {\bar{2}}_{-}|{\bar{2}}_{-}\rangle }_{N}=1$ and $C_{2}$ is defined in (25). The result of the deformation of state (43) is thus the deformed Werner state of two indistinguishable qubits $\rho_{D}\left(t\right)={\bar{W}}_{D}^{-}\left(t\right)$ [55], where

(46) $$\begin{array}{cc} {\bar{W}}_{D}^{-}\left(t\right) & :=[\left(1-\frac{3}{4}\;p\left(t\right)\right)\;C_{1}^{2}\;{|{\bar{1}}_{-}\rangle }_{N}{\langle {\bar{1}}_{-}|}_{N}\\ & \;+C_{2}^{2}\;\;\frac{1}{4}\;p\left(t\right)\;\left({|{\bar{1}}_{+}\rangle }_{N}{\langle {\bar{1}}_{+}|}_{N}+{|{\bar{2}}_{+}\rangle }_{N}{\langle {\bar{2}}_{+}|}_{N}+{|{\bar{2}}_{-}\rangle }_{N}{\langle {\bar{2}}_{-}|}_{N}\right)]\\ & \;/\left[1-\eta \;{\left|\langle \psi_{1}|\psi_{2}\rangle \right|}^{2}\left(1-\frac{3}{2}\;p\left(t\right)\right)\right]. \end{array}$$

To perform the final sLOCC measurement we assume that $|\psi_{1}\rangle ,\;|\psi_{2}\rangle$ have the usual structure given in Equation (10). Applying the projection operator on the state (46) as defined in Equation (6) we obtain

(47) $$\begin{array}{cc} \rho_{\mathrm{LR}}\left(t\right) & =[\left(1-\frac{3}{4}\;p\left(t\right)\right)\;{\left|lr^{\prime }-\eta \;l^{\prime }r\right|}^{2}{|1_{-}\rangle }_{\mathrm{LR}}{\langle 1_{-}|}_{\mathrm{LR}}\\ & +\frac{1}{4}\;p\left(t\right)\;{\left|lr^{\prime }+\eta \;l^{\prime }r\right|}^{2}\left({|1_{+}\rangle }_{\mathrm{LR}}{\langle 1_{+}|}_{\mathrm{LR}}+{|2_{+}\rangle }_{\mathrm{LR}}{\langle 2_{+}|}_{\mathrm{LR}}+{|2_{-}\rangle }_{\mathrm{LR}}{\langle 2_{-}|}_{\mathrm{LR}}\right)]\\ & /\left[\left(1-\frac{3}{4}\;p\left(t\right)\right){\left|lr^{\prime }-\eta \;l^{\prime }r\right|}^{2}+\frac{3}{4}\;p\left(t\right)\;{\left|lr^{\prime }+\eta \;l^{\prime }r\right|}^{2}\right]. \end{array}$$

Before computing the concurrence we notice that, as for the phase damping channel, the state of Equation (47) is real and diagonal on the Bell states basis, thus being invariant under the localized action of the Pauli matrices $\sigma_{y}^{L}⊗\sigma_{y}^{R}$. Therefore, the concurrence is evaluated in terms of the four eigenvalues of $\rho_{\mathrm{LR}}\left(t\right)$, namely

$$\begin{array}{c} \lambda_{A}\left(t\right)=\frac{\left(1-\frac{3}{4}\;p\left(t\right)\right){\left|lr^{\prime }-\eta \;l^{\prime }r\right|}^{2}}{\left(1-\frac{3}{4}\;p\left(t\right)\right){\left|lr^{\prime }-\eta \;l^{\prime }r\right|}^{2}+\frac{3}{4}\;p\left(t\right)\;{\left|lr^{\prime }+\eta \;l^{\prime }r\right|}^{2}},\\ \lambda_{B}\left(t\right)=\lambda_{C}\left(t\right)=\lambda_{D}\left(t\right)=\frac{\frac{1}{4}\;p\left(t\right)\;{\left|lr^{\prime }+\eta \;l^{\prime }r\right|}^{2}}{\left(1-\frac{3}{4}\;p\left(t\right)\right){\left|lr^{\prime }-\eta \;l^{\prime }r\right|}^{2}+\frac{3}{4}\;p\left(t\right)\;{\left|lr^{\prime }+\eta \;l^{\prime }r\right|}^{2}}. \end{array}$$

Considering once again fermions with real and positive coefficients $l,r,l^{\prime },r^{\prime }$, the concurrence has the expression

(48) $$C\left(\rho_{\mathrm{LR}}\left(t\right)\right)=\max\left\{0,\;\frac{\left(1-\frac{3}{2}\;p\left(t\right)\right)\left[{\left(lr^{\prime }\right)}^{2}+{\left(l^{\prime }r\right)}^{2}\right]+2ll^{\prime }rr^{\prime }}{{\left(lr^{\prime }\right)}^{2}+{\left(l^{\prime }r\right)}^{2}+\left(1-\frac{3}{2}\;p\left(t\right)\right)2ll^{\prime }rr^{\prime }}\right\}.$$

**Figure 10:**
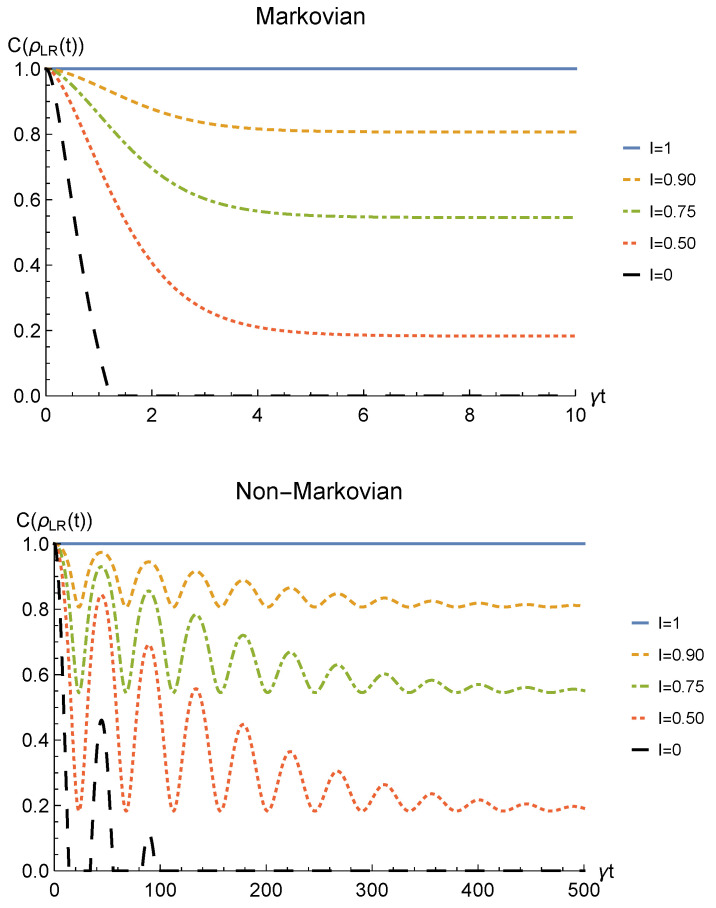
Concurrence of two identical qubits (fermions with $l,l^{\prime },r,r^{\prime }>0$, bosons with one of these four coefficients negative) in the initial state ${|1_{-}\rangle }_{\mathrm{AB}}$ subjected to localized depolarizing channels, undergoing an instantaneous deformation + sLOCC operation at time *t* for different degrees of spatial indistinguishability I (with $\left|l|=\right|r^{\prime }|$). Both Markovian (λ=5γ) (upper panel) and non-Markovian (λ=0.01γ) (lower panel) regimes are reported.

**Figure 11:**
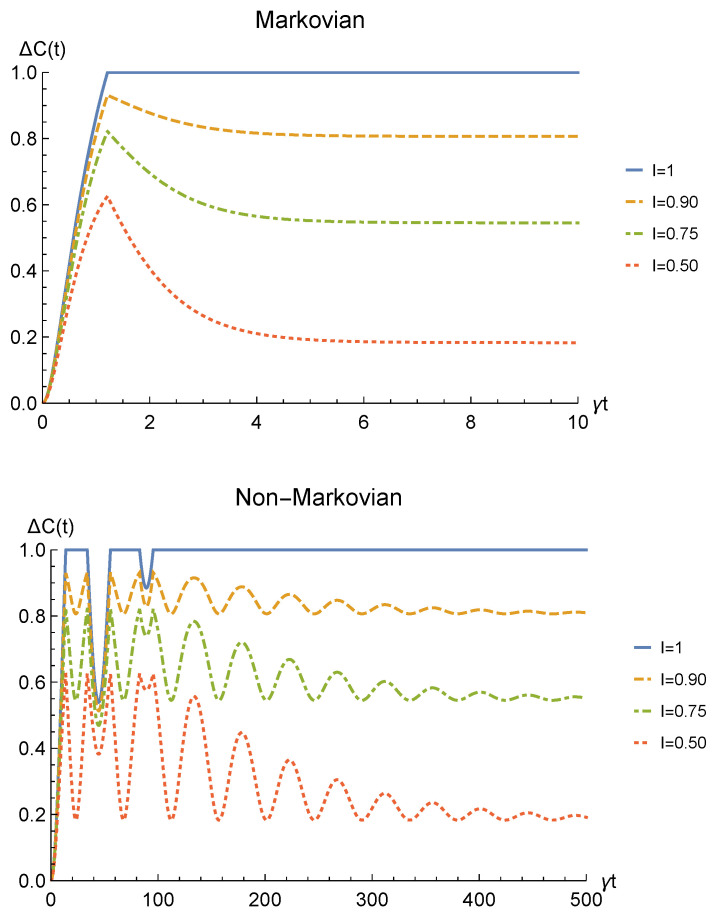
Net gain in the entanglement recovery of two identical qubits (fermions with $l,l^{\prime },r,r^{\prime }>0$, bosons with one of these four coefficients negative) in the initial state ${|1_{-}\rangle }_{\mathrm{AB}}$ interacting with a depolarizing channel, thanks to the deformation+sLOCC operation performed at time *t*. Results are reported for different degrees of spatial indistinguishability I (with $\left|l|=\right|r^{\prime }|$). Both the Markovian (λ=5γ) (upper panel) and non-Markovian (λ=0.01γ) (lower panel) regimes are shown.

Figure 10 shows the time behavior of entanglement quantified by Equation (48), while Figure 11 depicts ΔC(t). First of all, we emphasize that, differently from the amplitude damping channel and the phase damping channel, a sudden death phenomenon occurs when no deformation and sLOCC are performed: indeed, when I=0 (black dashed line) the entanglement vanishes at the finite time $\tilde{t}$ such that $p(\tilde{t})=2/3$. However, when 0<I<1, the state emerging from the sLOCC procedure recovers an amount of entanglement which decreases monotonically with *t* in the Markovian regime and periodically in the non-Markovian regime. Nonetheless, as in the phase damping case, such decrease approaches a constant value given by

(49) $$C_{\infty }=\max\left\{0,\;-\frac{{\left(lr^{\prime }\right)}^{2}+{\left(l^{\prime }r\right)}^{2}-4\;ll^{\prime }rr^{\prime }}{2\left[{\left(lr^{\prime }\right)}^{2}+{\left(l^{\prime }r\right)}^{2}-ll^{\prime }rr^{\prime }\right]}\right\}.$$

Furthermore, we notice once again that when the maximum spatial indistinguishability (I=1, blue solid line) is achieved, our procedure allows for a complete entanglement recovery independently on *t*.

As a further quantity of interest we obtain the sLOCC probability of success, defined in Equation (7), for two identical qubits whose correlations have been spoiled by a local depolarizing channel, that is

(50) $$P_{\mathrm{LR}}\left(t\right)=\frac{{\left(lr^{\prime }\right)}^{2}+{\left(l^{\prime }r\right)}^{2}-2\eta \;ll^{\prime }rr^{\prime }\left(1-\frac{3}{2}\;p\left(t\right)\right)}{1-\eta \left[{\left(ll^{\prime }\right)}^{2}+{\left(rr^{\prime }\right)}^{2}+2ll^{\prime }rr^{\prime }\right]\left(1-\frac{3}{2}\;p\left(t\right)\right)}.$$

**Figure 12:**
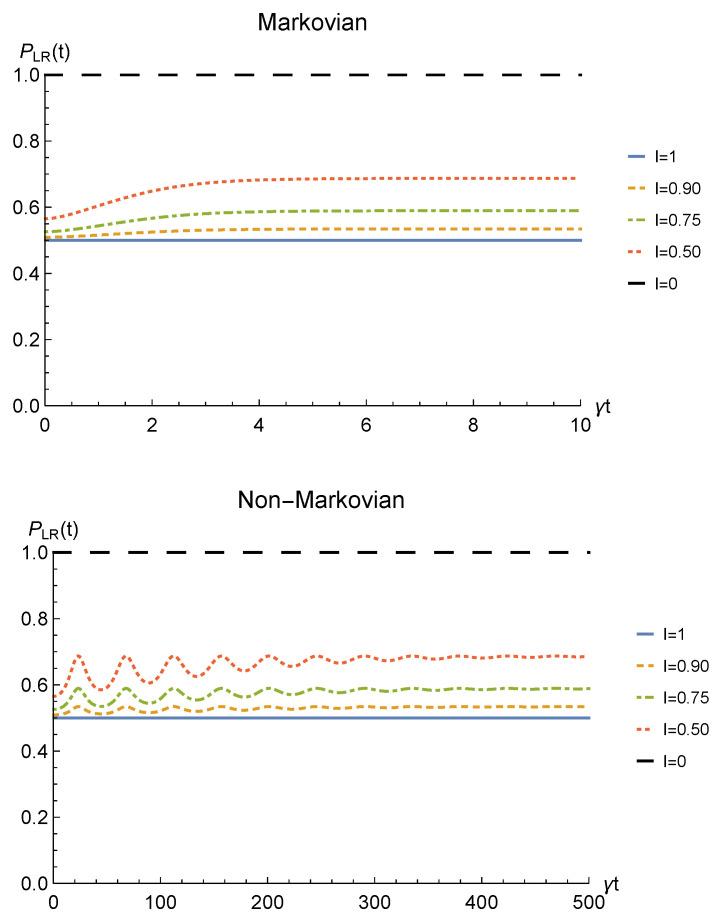
Probability of obtaining a nonzero outcome from the sLOCC projection for fermions with real and positive coefficients ($l=r^{\prime }$) under a depolarizing channel. Different degrees of spatial indistinguishability I are reported in both Markovian (λ=5γ) (upper panel) and non-Markovian (λ=0.01γ) (lower panel) regimes.

In Figure 12, $P_{\mathrm{LR}}\left(t\right)$ is plotted in the case of two fermions (with real and positive coefficients and $l=r^{\prime }$) for different degrees of spatial indistinguishability. Again, as expected, a trade-off exists between the probability of success and the concurrence, with the higher probability achieved when the qubits are perfectly distinguishable. Nonetheless, as happens in the previous channels, such probability reaches a stationary value that decreases as the indistinguishability increases, with $P_{\mathrm{LR}}=1/2$ as the minimum value when I=1 (blue solid line). For bosons, a similar behavior is found (with the constraint $l=r^{\prime }=l^{\prime }=-r$), having $P_{\mathrm{LR}}\left(t\right)=1-3p\left(t\right)/4$ when I=1 [55].

**Figure 13:**
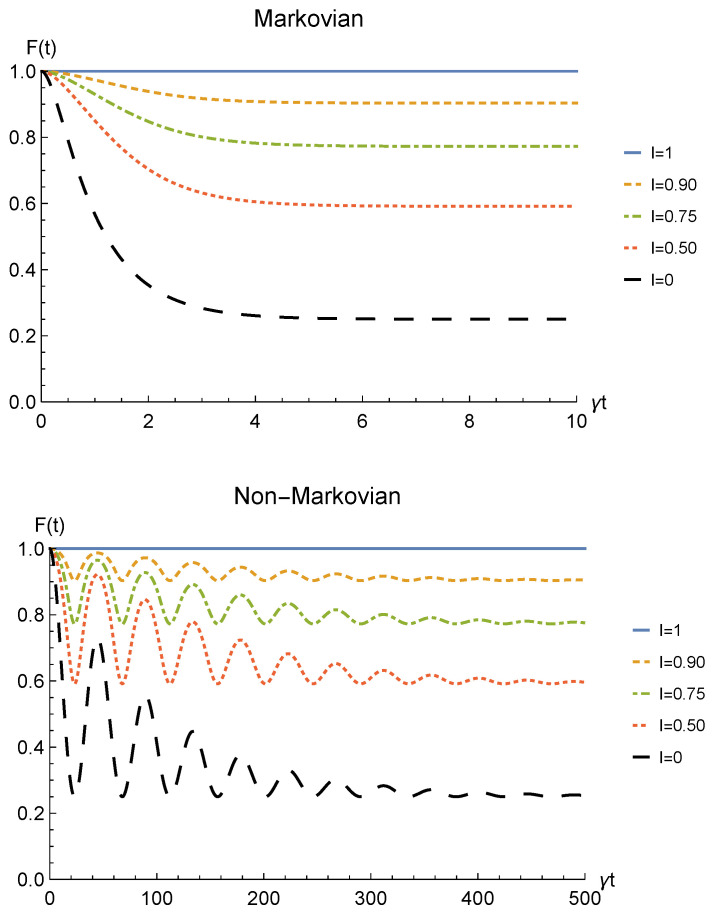
Fidelity of two identical qubits (fermions with $l,l^{\prime },r,r^{\prime }>0$) subjected to localized depolarizing channels, computed between the initial state ${|1_{-}\rangle }_{\mathrm{LR}}{\langle 1_{-}|}_{\mathrm{LR}}$ and the state produced by an instantaneous deformation+sLOCC operation at time *t* for different degrees of spatial indistinguishability I (with $\left|l|=\right|r^{\prime }|$). Both the Markovian (λ=5γ) (upper panel) and non-Markovian (λ=0.01γ) (lower panel) regimes are reported.

Finally, we show in Figure 13 the fidelity between the initial state $\rho_{0}={|1_{-}\rangle }_{\mathrm{LR}}{\langle 1_{-}|}_{\mathrm{LR}}$ and the final one (47), for fermions with real and positive coefficients and different degrees of spatial indistinguishability.

Once again, spatial indistinguishability is found to be directly influencing the fidelity, with a general behavior identical to the one emerged for the phase damping channel (Figure 9). Nonetheless, the asymptotic value reached for distinguishable particles in this scenario is F=1/4 (I=0, black dashed line), placed in between the two other channels considered in this work. State robustness is eventually achieved by the proposed procedure.

## 4. Discussion

In this paper we have shown that spatially localized operations and classical communication (sLOCC) provide an operational framework to successfully recover the quantum correlations between two identical qubits spoiled by the independent interaction with two noisy environments. The performance of such procedure is found to be strictly dependent on the degree of spatial indistinguishability reached by the spatial deformation of the particles wave functions. A general behavior has emerged: the higher is the degree of spatial indistinguishability, the better is the efficacy of the protocol, quantified by the difference between the amount of entanglement present at time *t* with and without the application of our procedure. In particular, when the two particles are brought to perfectly overlap and the maximum degree of indistinguishability is achieved, the initial (maximum) amount of entanglement is completely recovered in all the considered scenarios, independently of how long the qubits have been interacting with the detrimental environment.

If the indistinguishability is not maximum, instead, our results show that for an amplitude damping channel-like environment the entanglement after the sLOCC drops to zero after a short interaction time; nonetheless, the interval of time where the amount of recovered entanglement is significant increases with the indistinguishability in both the Markovian and the non-Markovian regimes. When the environment acts as a phase damping channel, instead, the recovered correlations are always nonzero and our protocol provides an exploitable resource independently of the interaction time (stationary entanglement). This behavior also holds in the depolarizing channel scenario, where the *deformation+sLOCC protocol* achieves a special usefulness since it allows us to recover quantum correlations destroyed at finite time by a sudden death phenomena.

Analogous characteristics are found also for the fidelity between the initial pure state and the post-processing state produced by the *deformation+sLOCC* protocol: when the indistinguishability is maximum, such quantity maintains its maximum value constant. When I<1, instead, it drops to zero faster as the indistinguishability decreases for an amplitude damping-like environment, while it reaches a constant value that grows with the indistinguishability when a phase damping channel or a depolarizing channel are considered. State robustness is therefore achieved.

We point out that the results reported in Figure 2, Figure 6 and Figure 10 show a similar behavior to the ones discussed in Reference [63] (for a Markovian regime) where, in contrast to the present analysis, the system–environment interaction occurs between the deformation bringing the particles to spatially overlap and the final sLOCC measurements. Nonetheless, the decay rate is much larger in the situation considered here: the sLOCC operational framework for entanglement recovery performs better when the environment is not able to distinguish the particle it is interacting with, as happens in Reference [63]. Despite this, the present protocol deals with a different physical context: indeed, it expressly refers to the scenario where we are given a two identical particles with entangled states that were spoiled by the environment in a situation where the particles remain distinguishable. Furthermore, in a real world application it is likely that the system–environment interaction will occur both before the (spatial) deformation and between the deformation and the sLOCC. Therefore, an interesting possible prospect of this work would be to investigate the general open quantum system framework provided in Reference [63] when applied to noisy initial states such as those given in Equations (21), (33) and (43).

Our results can apply to all the physical systems made of identical particles undergoing the noisy interactions discussed in Section 3, e.g., Bose–Einstein condensates, cavity and circuit QED systems and quantum photonics, once we are experimentally able to implement the deformation + sLOCC procedure. Among these scenarios, quantum photonics is most likely the best candidate for a first experimental verification of our results; indeed, in Reference [56] the authors have managed to experimentally apply the deformation + sLOCC protocol to a pair of photons in a tunable way using a simple optical setup. It is expected that such setup may be used as a starting point to validate the results discussed in this paper, where the implementation of simulated noisy environments is a task that can be easily achieved using linear optics devices [79].

Our findings ultimately provide further insights about protection techniques of entangled states from the detrimental effects of surrounding environments by suitably manipulating the inherent indistinguishability of identical particle systems.

## Data Availability

Not applicable.
